# MACE RNA sequencing analysis of conjunctival squamous cell carcinoma and papilloma using formalin-fixed paraffin-embedded tumor tissue

**DOI:** 10.1038/s41598-020-78339-6

**Published:** 2020-12-04

**Authors:** Stefaniya Boneva, Anja Schlecht, Peipei Zhang, Daniel Boehringer, Thabo Lapp, Hans Mittelviefhaus, Thomas Reinhard, Claudia Auw-Haedrich, Guenther Schlunck, Julian Wolf, Clemens Lange

**Affiliations:** Eye Center, Medical Center, Faculty of Medicine, University of Freiburg, Killianstrasse 5, 79106 Freiburg, Germany

**Keywords:** Preclinical research, Transcriptomics

## Abstract

Recent advances in the field of biomedical research allow for elucidation of the transcriptional signature of rare tumors such as conjunctival squamous cell carcinoma (SCC). In this study we compare its expression profile to conjunctival papilloma (Pap) and healthy conjunctival tissue (Ctrl) and develop a classification tool to differentiate these entities. Seven conjunctival SCC, seven Pap and ten Ctrl were formalin-fixed and paraffin-embedded (FFPE) and analyzed using Massive Analysis of cDNA Ends (MACE) RNA sequencing. Differentially expressed genes (DEG) and gene ontology (GO) clusters were explored and the abundance of involved cell types was quantified by xCell. Finally, a classification model was developed to distinguish SCC from Pap and Ctrl. Among the most prominent DEG in SCC a plethora of keratins were upregulated when compared to Pap and Ctrl. xCell analysis revealed an enrichment of immune cells, including activated dendritic cells and T-helper type 1 cells (Th1), in SCC when compared to Ctrl. The generated classification model could reliably discriminate between the three entities according to the expression pattern of 30 factors. This study provides a transcriptome-wide gene expression profile of rare conjunctival SCC. The analysis identifies distinct keratins, as well as dendritic and Th1 cells as important mediators in SCC. Finally, the provided gene expression classifier may become an aid to the conventional histological classification of conjunctival tumors in uncertain cases.

## Introduction

Conjunctival squamous cell carcinoma (SCC) is the most common malignancy of the ocular surface with a worldwide reported incidence rate from 0.02 to 3.5 per 100,000^1^. Although SCC of the conjunctiva is generally considered a low-grade malignancy^2^, it can occasionally penetrate into adjacent tissues or migrate into the blood and lymphatic system^3^, in particular in immunosuppressed patients^4^ or patients with an abnormal immune condition such as atopic dermatitis^5–8^. Surgical excision of the tumor, sometimes requiring extensive resection or enucleation, in rare cases even exenteration, usually is curative. An important differential diagnosis of conjunctival SCC is conjunctival papilloma (Pap)^9^, which belongs to the most frequently acquired epithelial tumors of the conjunctiva^10^.

The etiology of conjunctival SCC and Pap is not clarified for the most part. Whereas the formation of Pap is strongly associated with the human papilloma virus (HPV) types 6 and 11, which induce proliferation of dermal connective tissue followed by acanthosis and hyperkeratosis^11^, infections with HPV type 16 and 18 or human immunodeficiency virus (HIV) are discussed as possible risk factors for SCC^12,13^. For SCC, UV radiation has further been postulated to induce DNA damage and mutations in the tumor suppressor gene p53^14,15^ and reactivate latent HPV and HIV infections^16,17^, thus increasing the risk for conjunctival SCC. Furthermore, failure of DNA repair mechanisms, a reduced immunity and uncontrolled cell replication, for example by downregulation of the 14-3-3σ protein in keratinocytes^18^, are currently discussed to contribute to SCC formation^12^. A recent microarray study sheds light into the etiology of conjunctival SCC^19^, however this study is limited by its dependence on the standard genome annotations required for probe design and possible cross-hybridization problems^20^. The exact cellular and molecular mediators and the signaling pathways involved in the development of SCC remain not fully understood and a targeted molecular therapy approach is therefore not available.

Transcriptome-wide gene expression analysis is currently evolving as an important tool in cancer research to reveal gene expression signatures and molecular diagnostic markers and to define potential therapeutic targets^21–23^. In light of these recent endeavors in the field of biomedical research, the aim of this study was to elucidate the cellular and molecular mediators of conjunctival SCC by comparing them to Pap and healthy conjunctiva and to define their expression signature as an aid to the histological classification. The transcriptional signatures of conjunctival SCC presented in this study may improve our understanding of the disease, as well as its diagnosis and eventually contribute to successful personalized therapeutic approaches.

## Methods

### Patients

A total of 24 conjunctival samples from 24 patients were included in this study. The tissue samples were collected during therapeutic procedures and not specifically for this study. Seven subjects with conjunctival SCC and seven subjects with Pap who had been treated at the Eye Center of the University of Freiburg from 2003 to 2017 were retrospectively enrolled in this study. Healthy conjunctival tissue samples (superior quadrants in proximity to the limbus) from ten subjects who underwent buckling or 20-gauge vitrectomy surgery for retinal detachment served as controls. All tissue samples were analyzed in an anonymized manner. Ethics Committee approval was granted from the Ethics Committee Freiburg for this study and a written informed consent was obtained from each patient. All experiments were performed in accordance with the standards of the Declaration of Helsinki.

### Paraffin embedding

Formalin fixation and paraffin embedding (FFPE) of tumor and control tissue were performed according to routine protocols as previously described^24^. Briefly, specimens were fixed immediately after surgery in 4% formalin for 12 h, dehydrated in alcohol and finally processed for paraffin embedding. Paraffinized tissue samples were then processed for RNA isolation. 4-µm-thick sections were cut, mounted on silanized slides and deparaffinized in xylol-alcohol. Following routine histological staining, the histological diagnosis for each specimen was determined by an experienced ophthalmic pathologist.

### RNA isolation

Total RNA was isolated from ten to fifteen formalin-fixed and paraffin-embedded (FFPE) 4-µm-thick sections from each specimen using the Quick-RNA FFPE Kit (Zymo Research, USA). Following a DNAse I digestion using the Baseline-ZERO kit (Epicentre, USA), the RNA concentration was measured with the Qubit RNA HS Assay Kit on a Qubit Fluorometer (Life Technologies, USA). The RNA quality was determined with the RNA Pico Sensitivity Assay on a LabChip GXII Touch (PerkinElmer, USA).

### MACE libraries

RNA sequencing was performed using massive analysis of cDNA ends (MACE), a 3′ RNA sequencing method. The preparation of MACE libraries was carried out using 1 µg of total RNA, as previously described^25^. The barcoded libraries (seven SCCs, seven Pap and ten healthy conjunctival samples) were sequenced on the NextSeq 500 (Illumina, USA) with 1× 75 bp. PCR bias was removed using unique molecular identifiers. The sequence data have been submitted to the Gene Expression Omnibus database under accession number GSE147449.

### Statistics and bioinformatics

Sequencing data were uploaded to and analyzed on the Galaxy web platform (usegalaxy.eu)^26^, as previously described^27^. Quality control was performed with *FastQC Galaxy Version 0.72*^28^*.* Reads were mapped to the human reference genome (Gencode, release 31, hg38) with *RNA STAR Galaxy Version 2.7.2b*^29^ with default parameters using the Gencode annotation file (Gencode, release 31, https://www.gencodegenes.org/human/releases.html). Reads mapped to the human reference genome were counted using *featureCounts Galaxy Version 1.6.4*^30^ with default parameters using the aforementioned annotation file. The output of featureCounts was imported to RStudio (Version 1.2.1335, R Version 3.5.3). Gene symbols and gene types were determined based on ENSEMBL release 97 (July 2019) (Human genes, GRCh38.p12, download on 09/28/2019)^31^. Genes with 0 mean reads in at least one group were removed from analysis. Batch effects were removed by the *limma* function *removeBatchEffect*^32^. After principal component analysis, differential gene expression with Benjamini–Hochberg adjusted (adj.) *p*-values was analyzed using the R package DESeq2 Version 1.22.2^33^ with default parameters. Transcripts with log2 fold change (log2 FC) > 2 or <  − 2, adjusted *p*-value < 0.05 and mean of normalized reads > 10 in the respective upregulated group were considered as differentially expressed genes (DEG). Heatmaps were created with the R package *ComplexHeatmap 1.20.0*^34^. Venn diagrams were created using the *VennDiagram* package^35^. Other data visualization was performed using the *ggplot2* package^36^. Gene enrichment analysis and its visualization were done using the R package *clusterProfiler 3.10.1*^37^. To elucidate the cellular profile within the normal conjunctival, Pap and SCC microenvironment, we applied the computational method xCell, which quantifies the abundance scores of 64 immune and stroma cell types including hematopoietic progenitors, epithelial cells, extracellular matrix cells as well as adaptive and innate immune cells^38^. For this purpose, transcripts per million were calculated based on the output of featureCounts (assigned reads and feature length), as previously described^39^. Enrichment scores were compared between different groups using Mann–Whitney U test. Cell types with p < 0.05 were considered to be significantly differentially enriched.

For each group, specific genes were identified based on the overlap of upregulated factors in comparison to the two other groups. The 10 highest expressed genes in each group were used to develop a 30-gene classifier, as previously described^40^. Briefly, Pearson correlation was calculated for each sample’s expression of these 30 genes to the mean expression of each group using leave one out validation^41,42^. Thus, the expression profile of the leave one out sample was compared to the remaining samples of each group, repeating the procedure for each sample. For example, a higher Pearson correlation with SCC mean expression than with Papilloma mean expression predicted the sample to be SCC. Each sample’s correlation pair was plotted (SCC vs. Pap and Pap vs. Ctrl). The distance of each point from the diagonal with slope 1 defined a score to differentiate between SCC and Pap or between Pap and healthy conjunctiva, respectively. Cutoff values were determined based on standard deviation of absolute values of a group’s distance.

## Results

**Figure 1:**
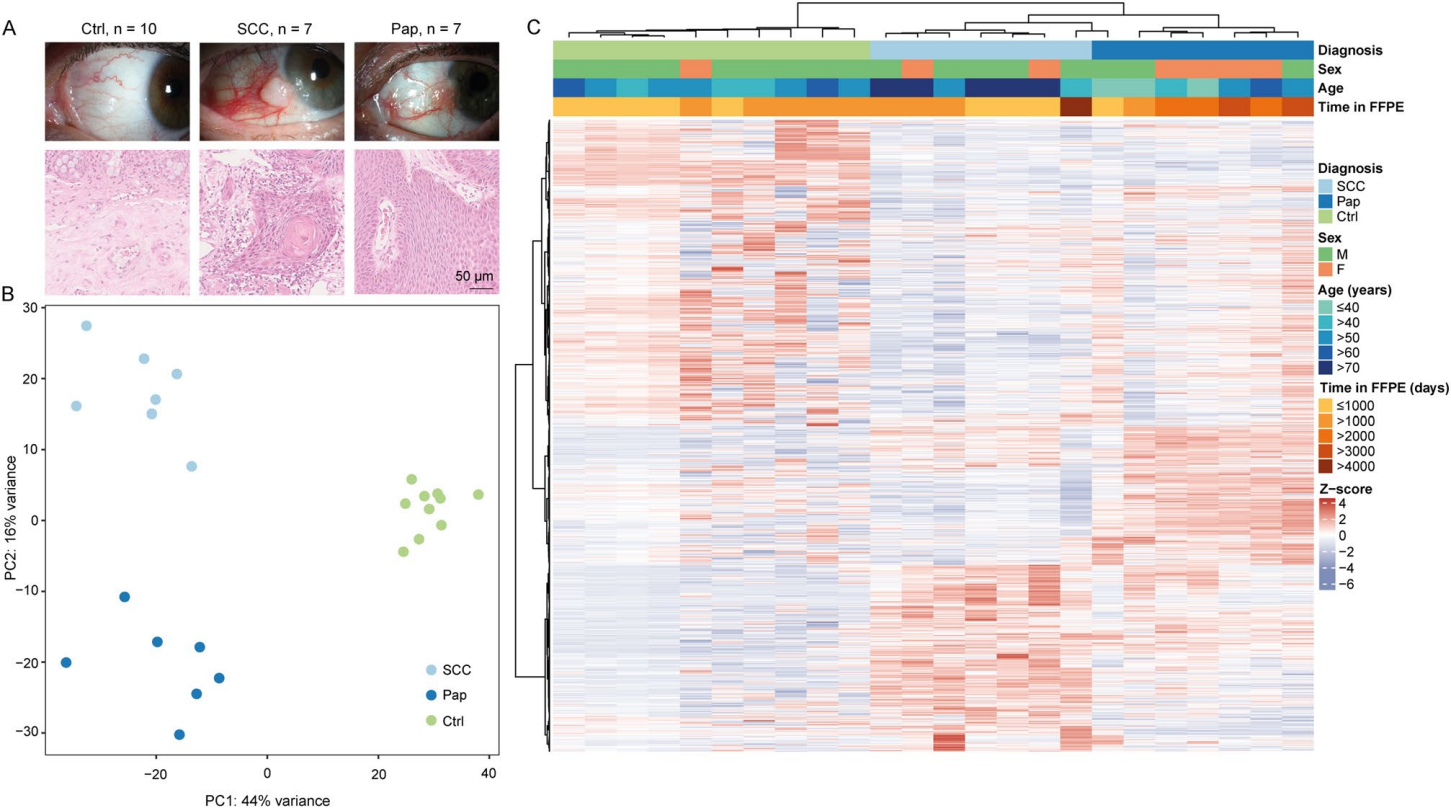
The transcriptional profiles of conjunctival squamous cell carcinoma (SCC), conjunctival papilloma (Pap) and healthy conjunctival samples (Ctrl) differ significantly. (**A**) Upper panel: Representative slit lamp pictures of a healthy conjunctiva (left-hand side panel), a conjunctival SCC (middle panel) and a conjunctival Pap (right-hand side panel). Lower panel: respective hematoxylin and eosin staining of the three analyzed entities. (**B**) Principal component analysis (PCA) plot illustrating the distribution of the three analyzed groups. (**C**) Heatmap clustering of all differentially expressed genes (DEG, log2 FC > 2 or < − 2 adjusted *p*-value < 0.05 and mean of normalized reads > 10 in the respective upregulated group). Color coding of the transcripts according to the z-score (deviation from a gene’s mean expression in standard deviation units). SCC, squamous cell carcinoma. Pap, Papilloma. Ctrl, healthy conjunctiva. M, male. F, female. FFPE, Formalin fixation and paraffin embedding.

24 conjunctival samples from 24 patients were included in this study—seven conjunctival SCC (five male and two female patients, mean age of 70 ± 13 years), seven conjunctival Pap (three male and four female patients, mean age of 37 ± 24 years) and ten healthy conjunctival tissue samples (nine male and one female patients, mean age of 55 ± 8 years) (Fig. 1A). Principal component analysis revealed samples clustering into three distinct groups, which co-segregated with clinical features of healthy conjunctiva, conjunctival papilloma and carcinoma (Fig. 1B). Differentially expressed genes between the three groups are illustrated in a heatmap, showing their clear segregation on a transcriptional level. No obvious sex- or time-in-FFPE-dependent influence on the transcriptional profile was found (Fig. 1C).

### Differential expression of genes in conjunctival SCC and Pap compared to healthy conjunctiva

**Figure 2:**
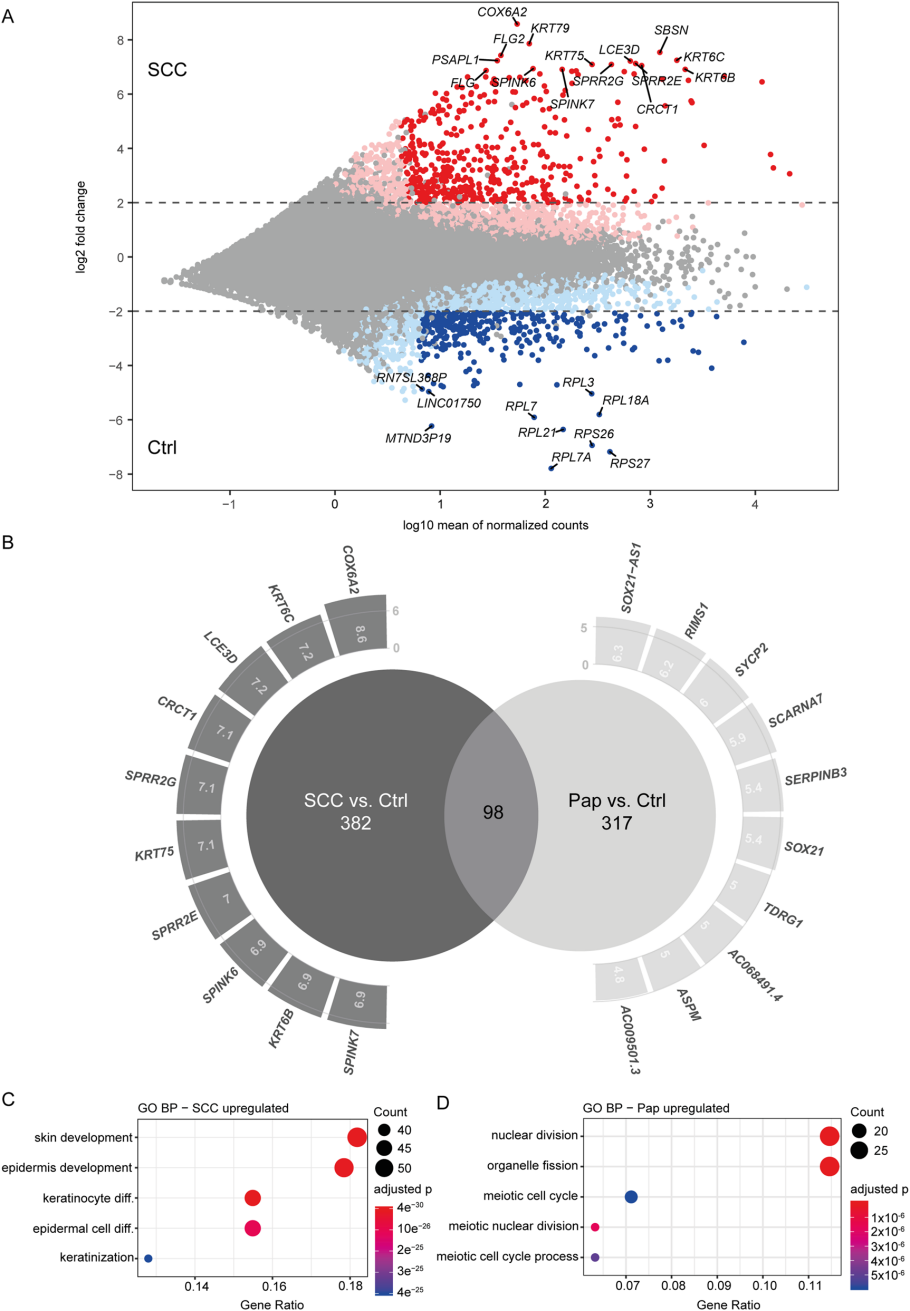
The transcriptional signatures of conjunctival SCC and conjunctival Pap differ significantly. (**A**) MA plot showing the differentially expressed genes in conjunctival SCC when compared to healthy conjunctiva. Light red dots represent factors with adjusted *p*-value < 0.05, while dark red dots are considered differentially expressed with additionally a log2FC > 2 and normalized reads > 10 in the SCC group. Light blue dots depict downregulated factors (adjusted *p*-value < 0.05), whereas dark blue dots are differentially downregulated (additionally log2FC < − 2 and normalized reads > 10 in the healthy conjunctival samples group). (**B**) A Venn diagram showing the transcripts expressed in both conjunctival SCC and Pap or specifically in one of both tumor groups. The most significantly expressed transcripts (top 10) are presented with their respective log2FC along the circumference of the Venn circles. Most significant SCC-specific (**C**) and Pap-specific (**D**) Gene ontology (GO) biological processes (BP) terms. The most enriched GO terms were chosen according to the adjusted *p* enrichment value. Within the graphic they are presented with the respective transcripts’ count (the size of the dot correlates to the count of factors within the GO term). The gene ratio describes the ratio of the count to the number of all DEG.

Next, we explored the differentially expressed genes in conjunctival SCC and Pap compared to conjunctiva. MACE analysis revealed 480 upregulated and 359 downregulated factors in SCC compared to healthy conjunctiva (Fig. 2A,B, SupplTbl 1 and 2). Among the 480 upregulated factors *COX6A2* (cytochrome c oxidase subunit 6A2), *KRT79* (keratin 79), *SBSN* (suprabasin), *FLG2* (filaggrin family member 2) and *KRT6C* (keratin 6C) were the most outstanding factors (Fig. 2A,B). The ribosomal proteins (*RP) RPL7A*, *RPS27*, *RPS26* and *RPL21* as well as the MT-ND3 pseudogene 19 (*MTND3P19*) were the most significantly downregulated RNAs in SCC compared to healthy conjunctiva (Fig. 2A). The comparison of conjunctival Pap to control tissue revealed 415 upregulated and 358 downregulated factors. *SOX21-AS1* (SOX21 antisense divergent transcript 1) was revealed as the most significantly upregulated gene and *MTCO2P12* (MT-CO2 pseudogene 12) as the most significantly downregulated one (Fig. 2B, SupplTbl 3 and 4). Next, we compared the differentially upregulated genes in SCC versus control tissue and in Pap versus control tissue (Fig. 2B). While 98 transcripts were upregulated in both SCC and Pap when compared to healthy conjunctiva, we detected 382 genes showing a specific overexpression in conjunctival SCC only (Fig. 2B). The GO terms with the most outstanding *p* enrichment value among this set of carcinoma-specific transcripts were *skin development*, *epidermis development* and *keratinocyte differentiation* (Fig. 2C). On the other hand we found 317 upregulated factors, which were exclusive in Pap and contributed to the GO terms *nuclear division, organelle splitting* and *meiotic cell cycle* (Fig. 2D).

**Figure 3:**
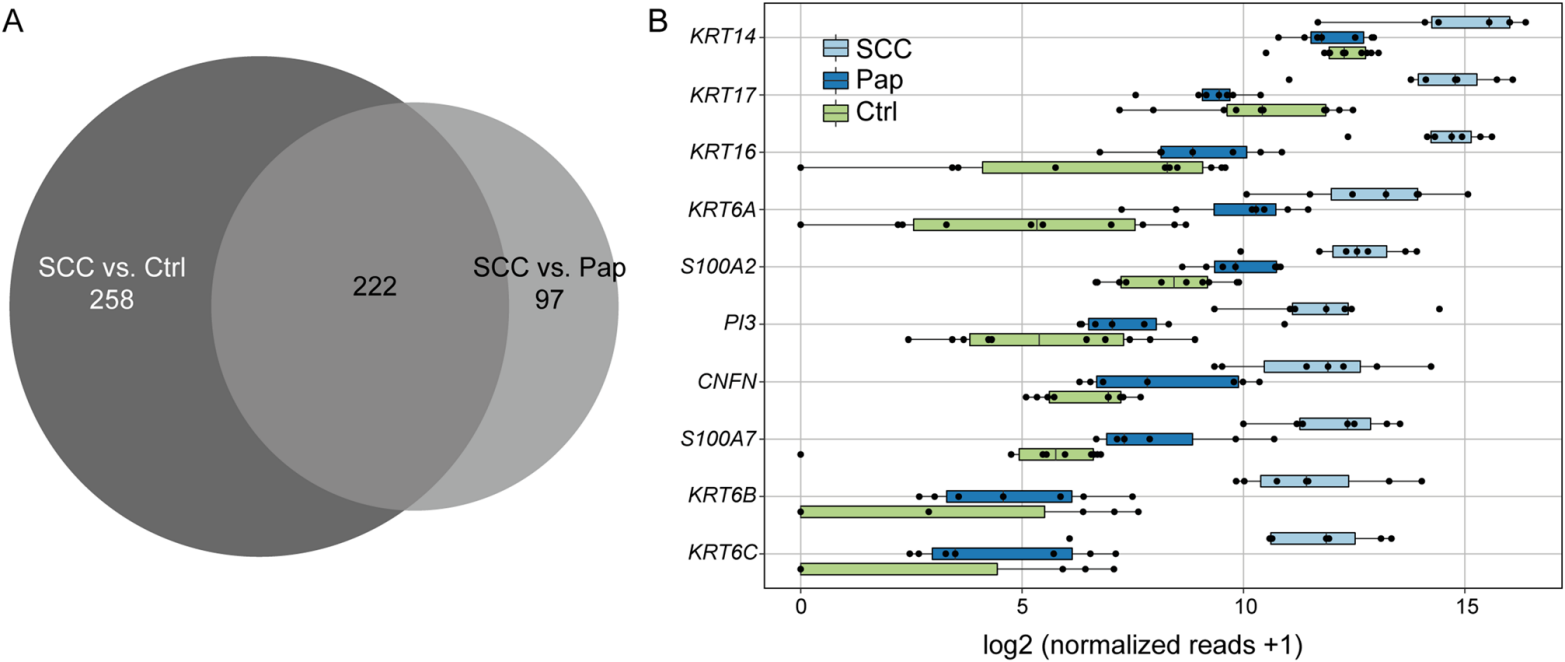
Conjunctival SCC express specific transcripts. (**A**) A Venn diagram showing the factors differentially expressed in SCC versus both Pap and control tissue revealing 222 SCC-specific transcripts. (**B**) Boxplots of the top 10 SCC-specific factors, listed according to the mean of normalized reads in SCC. SCC, squamous cell carcinoma. Pap, Papilloma. Ctrl, healthy conjunctiva.

To further determine SCC-specific factors we compared the differentially expressed genes in SCC versus control tissue and in SCC versus papilloma. We found 222 factors to be upregulated in SCC when compared to both, healthy conjunctiva and papilloma (Fig. 3A). The most significantly upregulated transcripts within this group (according to the mean of normalized reads in SCC) were the keratins 14, 17 and 16 (*KRT14*, *KRT16* and *KRT17*) (Fig. 3B).

Papilloma-specific factors were also defined by comparing the differentially expressed genes in Pap versus SCC and Pap versus control tissue. 184 transcripts were upregulated in Pap when compared to both other entities. The highest expressed transcript within this group (according to mean of normalized reads in Pap) was *YTHDC1* (YTH domain containing 1) (SupplFig. 1 A,B).

### Cell type enrichment analysis in conjunctival tumors

**Figure 4:**
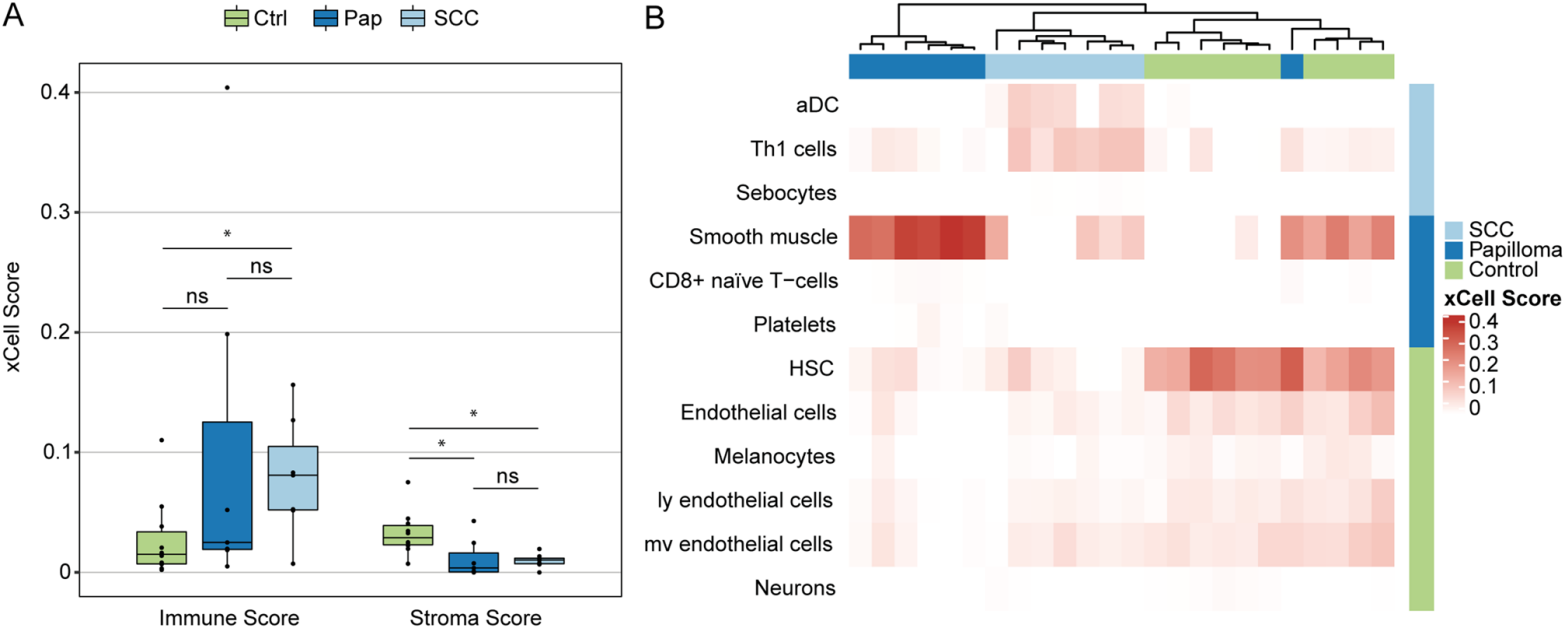
Cell type enrichment analysis in conjunctival SCC, Pap and healthy conjunctiva. (**A**) Boxplots of the xCell immune and stroma scores in SCC, Pap and healthy conjunctiva. Each dot represents one sample. *p < 0.05, ns: not significant (Mann–Whitney U test). (**B**) Heatmap with xCell scores of 12 specific cell types for SCC, Pap and healthy conjunctiva. Each row represents one cell type, each column represents one sample. Rows are ordered according to the fold change of mean enrichment scores for the respective up- and downregulated group, respectively. Columns are clustered according to similarities in xCell enrichment scores (see dendogram). aDC, activated dendritic cell. Th1, -helper type 1 T cells. HSC, hematopoietic stem cell. ly, lymphatic. mv, microvascular.

Since the RNA-Seq profile is a conglomerate of RNA of multiple cell types including resident and infiltrating cells, we first conducted cell type enrichment analysis to recover the identity of cell types found in conjunctival SCC, Pap and healthy conjunctiva, using the gene signature expression-based cell type enrichment tool xCell^38^. Cell type enrichment scores (ES) across 64 immune and stromal cell types were obtained for the three entities (Fig. 4A, SupplFig. 3). Our data demonstrates that immune scores representing the immune cell content were overall higher in SCC (0.08 ± 0.05) when compared to healthy conjunctiva (0.03 ± 0.03 for Ctrl, *p* = 0.04) and similar to Pap (0.1 ± 0.15, *p* = 0.52). The stromal scores, in contrast, were overall reduced in SCC (0.01 ± 0.01) and Pap (0.01 ± 0.02) when compared to healthy conjunctiva (0.03 ± 0.02; for SCC p = 0.004, for Pap, *p* = 0.03). Among the different cell subpopulations, activated dendritic cells (aDC) and T-helper type 1 cells (Th1) were significantly increased in SCC samples compared to both other groups (*p* < 0.05, Fig. 4B). In Pap, smooth muscle cells and CD8 + naïve T cells were significantly enriched in comparison to SCC and normal conjunctiva (*p* < 0.05), while control conjunctiva was characterized by an enrichment of hematopoietic stem cells and endothelial cells (*p* < 0.05, Fig. 4B).

### Classification model

**Figure 5:**
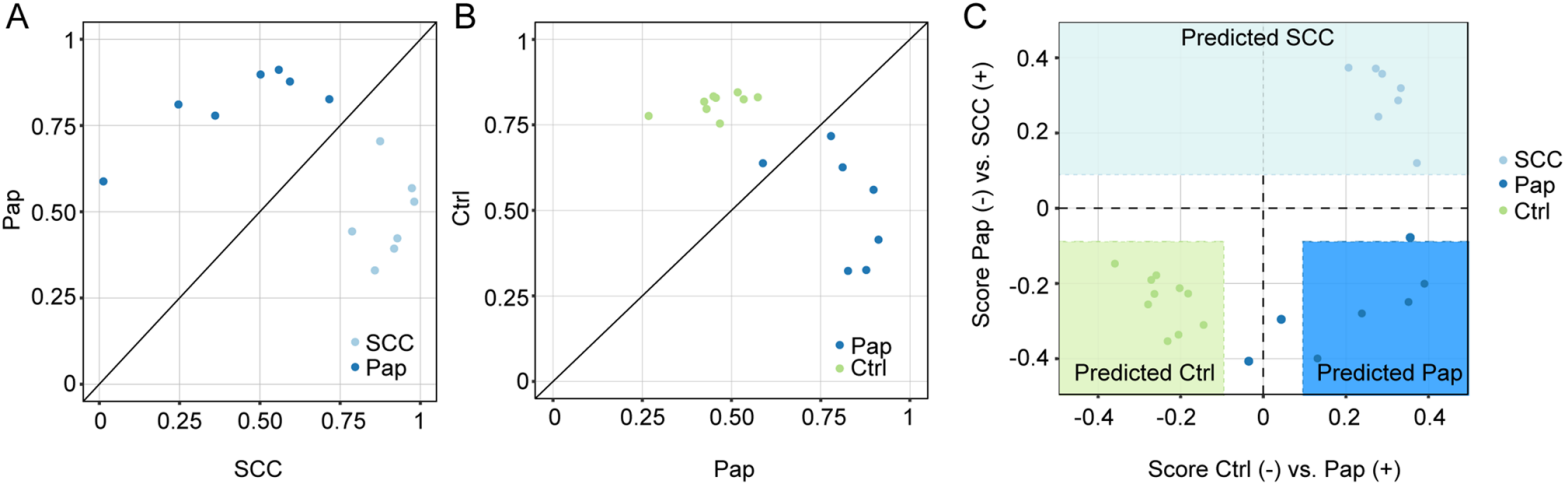
Classification model for conjunctival tumors. (**A**) Pearson values of the mean expression of the 30 specific genes in SCC vs. Pap (**A**) and Pap vs. Ctrl (**B**). (**C**) Classification score defined by the distance of each sample to the diagonal in (**A**) and (**B**). X-axis: comparison between Pap and Ctrl—a positive score points towards Pap, a negative score towards Ctrl. Y-axis: comparison between SCC and Pap—a positive score suggests SCC, a negative score—Pap. The colored fields illustrate the areas of significance. SCC, squamous cell carcinoma. Pap, Papilloma. Ctrl, healthy conjunctiva.

To build a classifier distinguishing SCC, Pap and normal conjunctiva, we developed a classification model on the basis of factors specific for the three analyzed groups. For this purpose, Pearson correlation was calculated for the mean expression of the top 10 SCC- (Fig. 3B), Pap- (SupplFig. 1) and Ctrl-specific transcripts (SupplFig. 2) to the mean expression of each group using leave one out validation (Fig. 5A,B).

The classification model showed a reliable classification of the diagnosis according to the expression pattern of the 30 genes (Fig. 5C). Using this approach, the sensitivity and specificity for discrimination of SCC, as well as for normal conjunctiva was 100.0% each. For Papilloma, the sensitivity and specificity were 57.1% and 100.0%, respectively. The overall accuracy of the model was 85.7% (AUC (area under the curve) for the whole model: 0.86).

## Discussion

Transcriptome-wide gene expression analysis is emerging as an important tool in cancer research to decipher altered gene expression patterns, detect molecular diagnostic markers and to define potential therapeutic targets. However, the transcriptional analysis of rare tumors, such as SCC, has been impeded in the past by their low incidence, which makes a prospective study with fresh tissue challenging. Formalin-fixed paraffin-embedded (FFPE) tissue samples of rare tumors, including conjunctival SCC and Pap, are, among others, routinely collected in clinical histological archives. The advent of 3′ RNA-Seq methods, such as the Massive Analysis of cDNA Ends (MACE) technology, allows for high throughput sequencing of FFPE specimens that have been stored in histological archives over the past decades^43,44^. In this study we conduct the first comparative analysis of the cellular tumor microenvironment and expression profiles of conjunctival SCC with conjunctival Pap and healthy conjunctiva using the novel MACE technique for sequencing archived samples paired with xCell data deconvolution for cell type discrimination in bulk transcriptomic data.

In line with histological observations of changed epithelial cell differentiation and keratinization^2^, our transcriptional analysis reveals a plethora of altered factors in SCC that contribute to biological GO processes such as *skin development, epidermis development* and *keratinocyte differentiation*. Almost the entire gene cluster of the epidermal differentiation complex (EDC) is cumulatively upregulated in conjunctival SCC when compared to healthy conjunctival tissue. The EDC comprises a gene complex of over fifty genes encoding molecules acting during the coordinated process of cornification, the specific form of differentiation and programmed cell death characteristic for keratinocytes^45^. Among them, e.g. *FLG2*, filaggrin family member 2, *LCE3D*, late cornified envelope 3D, *SPRR2G*, small proline rich protein 2G, and many more were upregulated in SCC in our dataset. The notion of altered differentiation in the malign tumor, as evidenced by our GO term analysis, is in contrast to the mere upregulation of proliferation-associated factors in the benign papilloma.

In accordance with the above-mentioned GO terms, we found several keratins (*KRT14*, *KRT17*, *KRT16*, *KRT6A*, *KRT6B*, *KRT6C*) to be significantly increased in conjunctival SCC when compared to both healthy conjunctiva and Pap. Changes in keratin subtype expression have been linked to various tumor species, particularly cutaneous epithelial tumors^46^, but also other squamous cell carcinoma types^40,47–50^. Their expression and organization have an impact on cell growth, migration and invasion, all hallmarks of cancer metastasis^51,52^. Furthermore, keratins function in immune recognition, tenacity to cellular stresses, posttranslational modifications that modulate intermediate filament assembly and cellular motility and thereby are highly relevant in cancer progression and tumor cell dissemination^53^. In line with our analysis SCC of the skin is characterized by the expression of the stratified epithelial keratins *KRT5* and *KRT14* and the hyperproliferative keratinocyte-type keratins *KRT6*, *KRT16* and *KRT17*, overexpressed upon cell stress and injury^54^. Furthermore, *KRT17* has been previously found to be upregulated in SCC of the conjunctiva in microarray analyses^19^. Taken together, this data supports the notion that stratified-epithelial keratins, in particular *KRT6* and *KRT17*, are useful as general markers for squamous cell carcinomas in histologically uncertain, poorly differentiated conjunctival SCC samples^54^.

In addition to keratins, S100 calcium-binding proteins (*S100A2* and *S100A7*) stand out as highly upregulated SCC-specific factors in our analysis. S100 family members are involved in a plethora of cellular processes including proliferation, differentiation, inflammation and apoptosis^55^. Altered S100A7 expression has been linked to poor clinical outcomes in several solid cancer types by promoting invasiveness of cancer cells via upregulation of MMPs^56^. Moreover, S100A2 is a crucial factor for proliferation and differentiation of epithelial cells^57^ and promoting neoplastic transformation^19^. Thus, S100A2 and S100A7 may possess certain prognostic and therapeutic potential in conjunctival SCC.

SCC are characterized by tissue infiltrating immune cells, epithelial cell differentiation and keratinization^2,58^. In line with these histological findings, the x-Cell-based bioinformatic cell type enrichment analysis demonstrated an overall increased immune cell score when compared to healthy conjunctiva. Among the different cell subpopulations, activated dendritic cells (aDC) and T-helper type 1 cells (Th1) were significantly increased in SCC samples compared to both other groups. These results concur with reports suggesting that the presence of dendritic cells correlates with poor prognosis for SCC of the ovary^59^ and SCC of the head and neck^60^. Dendritic cells are antigen presenting cells mediating a link between innate and adaptive immunity by secreting interleukin (IL)-12 and type I interferons and thus activating Th1 cells^61^, which are also substantially represented in conjunctival SCC in our cell type analysis. Thus, the accumulation of these immune cell types opens new perspectives for cell-specific treatment of conjunctival malignancies.

In order to define an expression signature as an aid to the histological classification of conjunctival papilloma and squamous cell carcinoma and to reduce potential observer bias and variability, we developed a classification model including 30 factors identified in our analysis as specific for the three entities (Ctrl, Pap and SCC) using a strategy described recently^40^. The model showed an overall accuracy of 0.86 (AUC), indicative of robust allocation. Thus, the presented RNA classifier provides an objective quantitative method to aid in the pathological diagnosis of conjunctival SCC and Pap. This non-subjective molecular classification of SCC and Pap may be useful in the future as an additional diagnostic tool for complicated histological cases and may serve to optimize the reproducibility and accuracy of the diagnosis. However, further studies featuring more patients and especially challenging cases (e.g. dysplastic changes in epithelium) are warranted to validate the discriminatory power of the classifier and confirm its applicability in clinical routine.

Although this study is retrospective, includes a limited sample size and relies on internal expression signature validation only (leave one out validation approach^41,42^), its findings are consistent with previous observations in conjunctival and cutaneous epithelial tumors, which supports this approach. In addition, a transcriptome-wide evaluation is inherently more comprehensive and serves as a basis for the generation of new hypotheses to be tested in the future. Single-cell RNA sequencing could provide a detailed impression of cell heterogeneity, but is not feasible with archived FFPE material. We employed cell type enrichment analysis in bulk RNAseq data using xCell^38^ to assess the identity of the major cell types found in conjunctival samples. It is important to note, that these results were not validated by histopathology due to the limited amount of available tissue and sample acquisition difficulties for a considerable number of immunohistochemical stainings. Further prospective studies with increased power are warranted for clinical validation of the developed classification model.

Taken together, the present study provides a high throughput transcriptome-wide gene expression profile of rare squamous cell carcinoma of the conjunctiva using the MACE technology in FFPE archival tumor samples. The analysis identifies, among others, dendritic cells and Th1 cells as primary accumulating cell types and keratins as highly expressed factors in SCC, which all together may contribute to tumor growth and disease progression. Finally, we developed a sensitive and specific gene expression classifier for conjunctival tumors that distinguishes SCC from Pap and normal conjunctiva, which may be useful as an additional diagnostic tool for complicated histological cases and serve to optimize the reproducibility and accuracy of the diagnosis.

## Supplementary Information

Supplementary Information 1.

Supplementary Information 2.

Supplementary Information 3.

Supplementary Information 4.

Supplementary Information 5.

## Data Availability

The sequence data have been submitted to the Gene Expression Omnibus database under accession number GSE147449. The password is available from the corresponding author upon request.
