# Supplementary material for: MACE RNA sequencing analysis of conjunctival squamous cell carcinoma and papilloma using formalin-fixed paraffin-embedded tumor tissue
Source: Sci Rep. 2020 Dec 4;10:21292. doi: 10.1038/s41598-020-78339-6 (PMC7718249; doi:10.1038/s41598-020-78339-6)
Supplement: Supplementary file 1 — Supplementary Information 1. [file 41598_2020_78339_MOESM1_ESM.pdf]

MACE RNA Sequencing Analysis of Conjunctival Squamous Cell Carcinoma and Papilloma Using Formalin-Fixed Paraffin-Embedded Tumor Tissue

Stefaniya Boneva[1], Anja Schlecht[1], Peipei Zhang[1], Daniel Boehringer[1], Thabo Lapp[1], Hans Mittelviefhaus[1], Thomas Reinhard[1], Claudia Auw-Haedrich[1], Guenther Schlunck[1], Julian Wolf[1] and Clemens Lange*[1]

[1]Eye Center, Medical Center, Faculty of Medicine, University of Freiburg, Germany

Corresponding author: Clemens Lange MD PhD, Eye Center, Medical Center – University of Freiburg, Killianstrasse 5, 79106 Freiburg Germany tel. +49 761 270 40511, fax +49 761 270 40630, e-mail: clemens.lange@uniklinik-freiburg.de

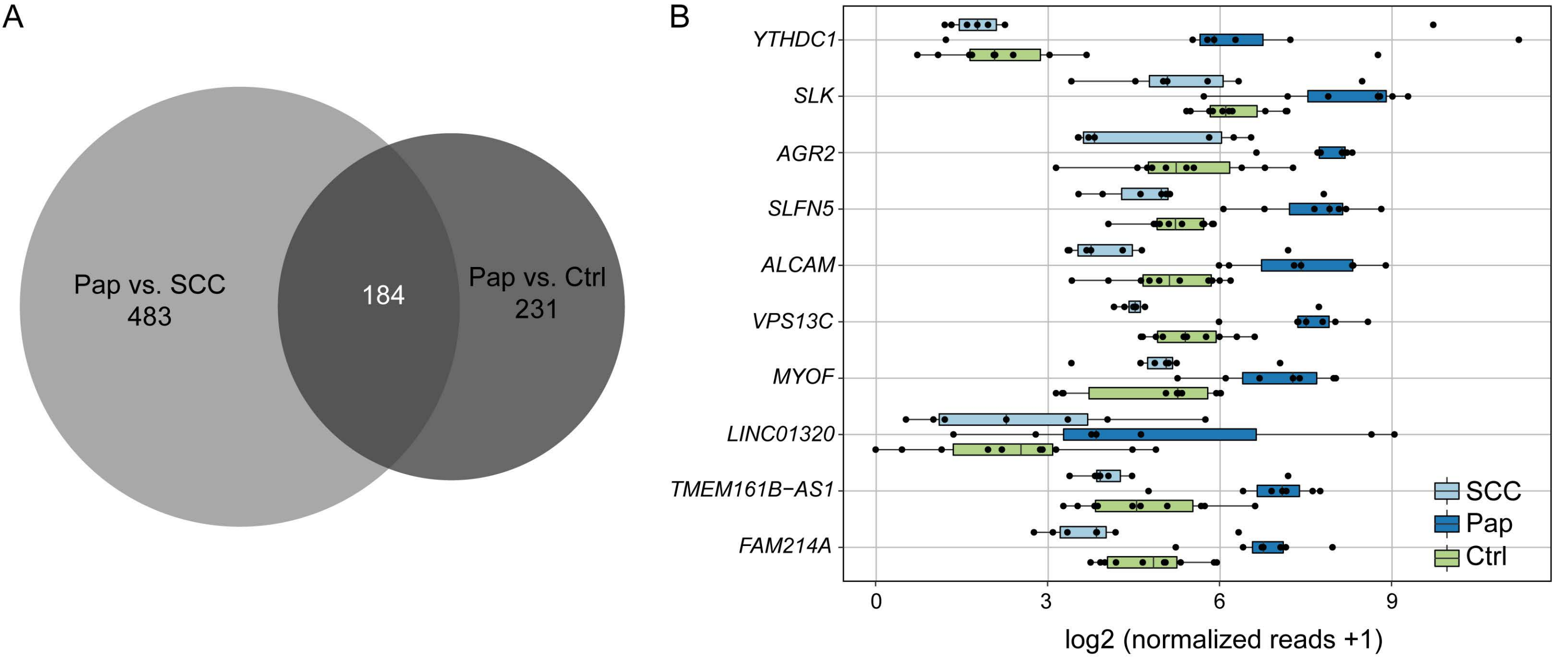

Supplementary Figure 1. Conjunctival Pap express specific transcripts. A) A Venn diagram showing the factors differentially expressed in Pap versus both SCC and control tissue. B) Boxplot of the top 10 Pap-specific factors (overlap from A), listed according to the mean of normalized reads in Pap. SCC, squamous cell carcinoma. Pap, Papilloma. Ctrl, healthy conjunctiva.

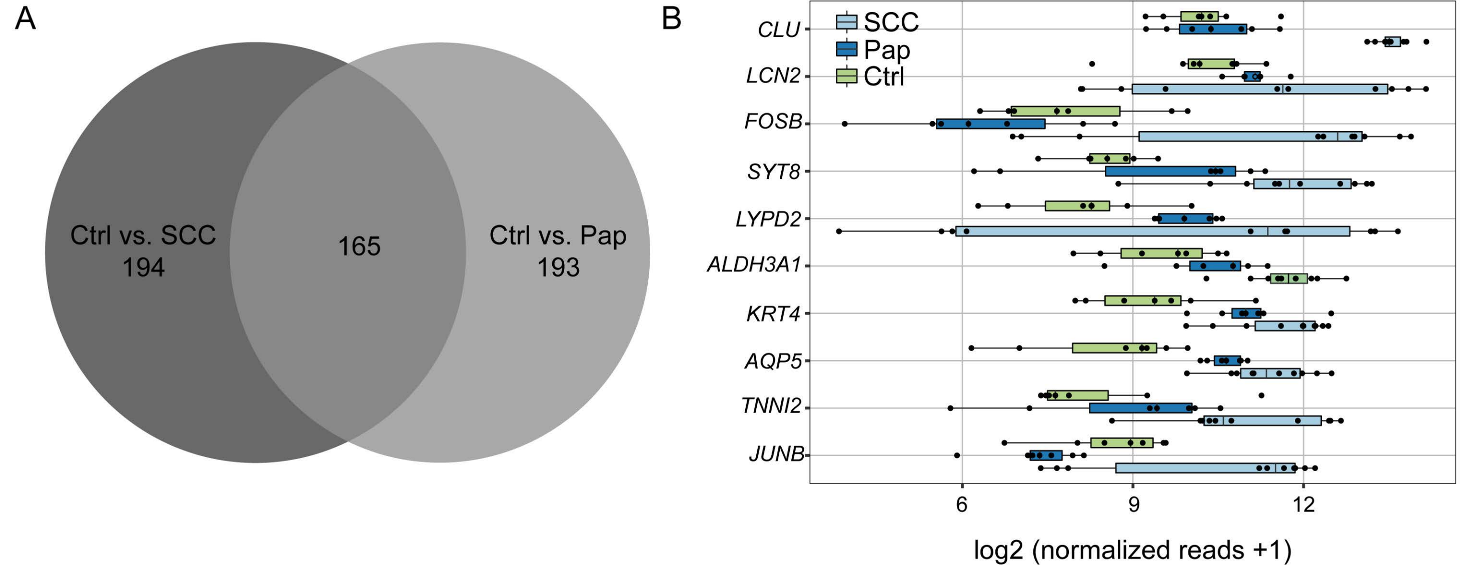

Supplementary Figure 2. Healthy conjunctival tissue expresses specific transcripts. A) A Venn diagram showing the factors differentially expressed in Ctrl versus both SCC and Pap. B) Boxplot of the top 10 Ctrl-specific factors (overlap from A), listed according to the mean of normalized reads in Ctrl. SCC, squamous cell carcinoma. Pap, Papilloma. Ctrl, healthy

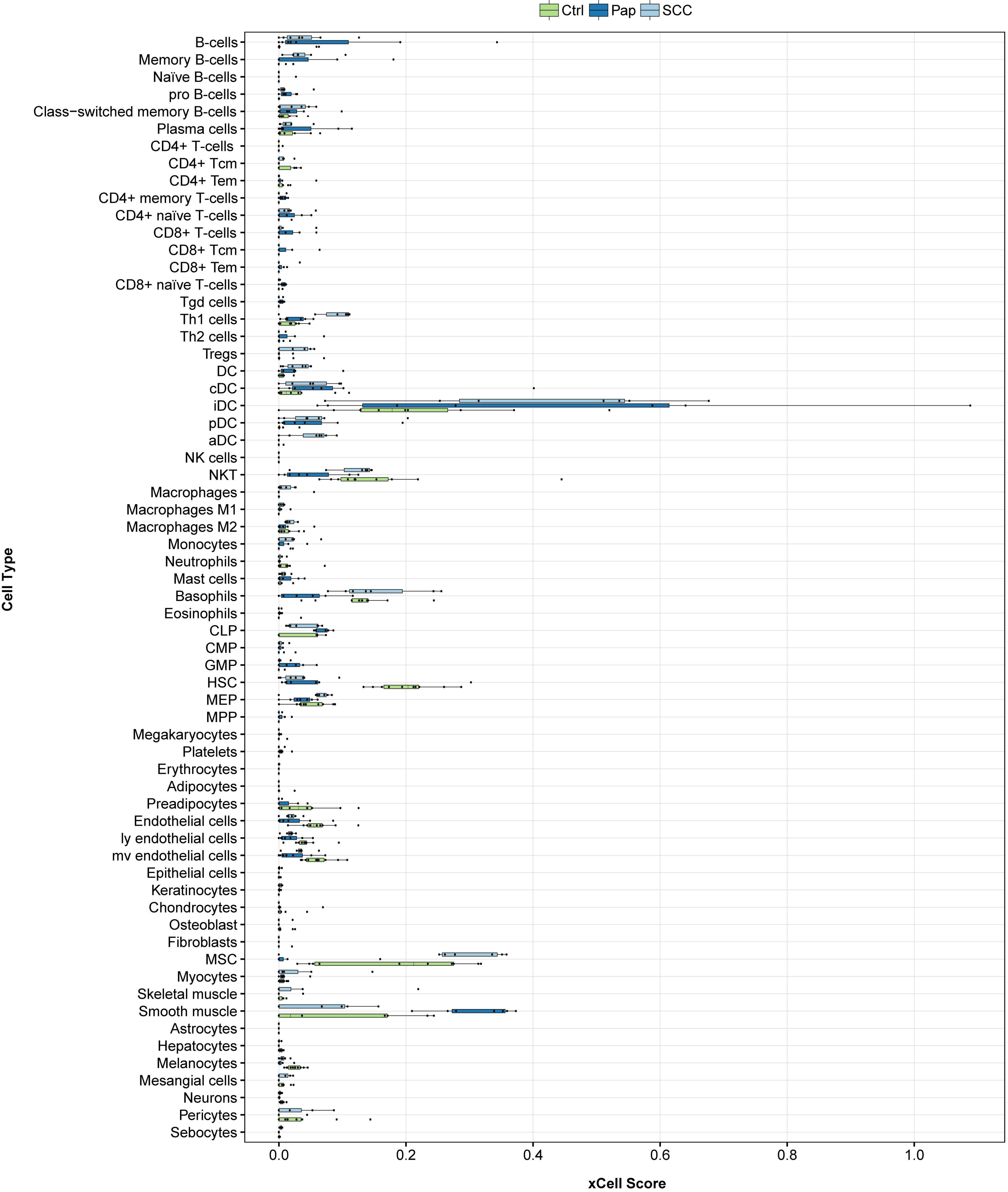

Supplementary Figure 3. Cell type enrichment analysis of bulk RNA sequencing data from conjunctival SCC, Pap and healthy conjunctiva. Cell type enrichment scores were generated via xCell based on gene expression data for 64 immune and stromal cell types. The x axis depicts the xCell enrichment score, and the y axis lists all 64 cell types analyzed. Each dot represents one sample. Immune cell types are presented at the top, whereas stromal cell types are shown in the lower half. Tcm, central memory T cell. Tem, effector memory T cell. Tgd cell, gamma delta T cell. Th1, T helper type 1. Th2, T helper type 2. Tregs, regulatory T cells. DC, dendritic cell. cDC, conventional dentritic cell. iDC, immature dentritic cell. pDC, plasmacytoid dendritic cell. aDC, activated dentritc cell. NK, natural killer. NKT, natural killer T cell. CLP, common lymphoid progenitor. CMP, common myeloid progenitor. GMP, granulocyte-monocyte progenitor. HSC, hematopoietic stem cells. MEP, megakaryocyte erythroid progenitor. MPP, multipotent progenitor. ly, lymphatic. mv, microvascular. MSC, mesenchymal stem cell.
